# Experiences and Therapy Needs of Parents With an Infant at High Risk for Development of Unilateral Spastic Cerebral Palsy: A Qualitative Interview Study

**DOI:** 10.1177/08830738251335052

**Published:** 2025-05-14

**Authors:** Cornelia H. Verhage, Maria J. C. Eijsermans, Madelon Kleingeld, Marjolijn Ketelaar, Jan Willem Gorter, Linda S. de Vries, Marco van Brussel, Agnes van den Hoogen

**Affiliations:** 1 Center for Child Development and Exercise, Wilhelmina Children's Hospital, University Medical Center Utrecht, Utrecht University, Utrecht, the Netherlands; 2 Department of Paediatric Psychology, Wilhelmina Children's hospital, University Medical Center Utrecht, Utrecht, the Netherlands; 3 De Hoogstraat Rehabilitation, Utrecht, Center of Excellence for Rehabilitation Medicine, Utrecht, the Netherlands; 4 Department of Rehabilitation, Physical Therapy Science and Sports, UMC Utrecht Brain Center, University Medical Center Utrecht, Utrecht, the Netherlands; 5 CanChild, Department of Pediatrics, McMaster University, Hamilton, Canada; 6 Department of Neonatology, Wilhelmina Children's Hospital, 8124 University Medical Center Utrecht, Utrecht University, Utrecht, the Netherlands

**Keywords:** cerebral palsy, developmental disability, infant, neonate, stroke

## Abstract

**Aim:** To understand experiences and therapy needs of parents with an infant with unilateral perinatal brain injury and at high risk for unilateral spastic cerebral palsy in the first year. **Patients and Methods:** Sixteen parents (from 8 children with unilateral spastic cerebral palsy, 3 without) diagnosed with unilateral perinatal brain injury participated in semistructured interviews. Data were analyzed using thematic analysis. **Results:** The overarching theme, “an unexpected journey,” included 4 subthemes: (1) “A roller coaster start”—stressful initial experiences on a neonatal intensive care unit; (2) “Wishing for a crystal ball”—need for information on (future) development; (3) “Reaching for the stars”—value of therapist guidance in supporting infant development; (4) “Growing seeds of confidence”—increased parental confidence in their child's development and their role. **Conclusion:** Parents have information needs about their child's (future) neurodevelopment. Physical or occupational therapists provide information, monitor motor progress, and guide parents in supporting development and can offer needed reassurance.

Cerebral palsy is the most common physical disability in childhood, which manifests early in life and has a prevalence of about 2 cases per 1000 live births.^1^ Forty percent of the total group of cerebral palsy comprises unilateral spastic cerebral palsy , which is the most common phenotype of cerebral palsy.^2^ Major advances in magnetic resonance imaging (MRI) combined with clinical assessments have resulted in a more accurate diagnosis of unilateral spastic cerebral palsy during the first year of life for many infants.^3^

In neonates with unilateral perinatal brain injury, neonatal MRI predicts cerebral palsy with a 86% to 100% sensitivity and 87% to 97% specificity, prior to clinical signs of unilateral spastic cerebral palsy.^4,5^ Detection of clinical signs of unilateral spastic cerebral palsy is vital for the diagnosis, but also for optimal neurodevelopmental outcome as the child benefits from starting intervention programs as early as possible.^3^ Furthermore, delays in diagnosis are associated with significant parental dissatisfaction, stress, and depression.^6-8^ When infants show disorders of movement or posture and a diagnosis of cerebral palsy cannot be made with certainty, it is recommended to use an interim clinical diagnosis of “high risk of cerebral palsy”^9^ and to start with pediatric physical and occupational therapy.^10^

Family-centered care is widely established in early intervention.^11^ Key in family-centered care is respect and dignity for family's perspectives, knowledge and characteristics, effective information shared by professionals to families, partnership, and collaboration.^12^ Family-centered care requires listening to families and being responsive to priorities. Several studies investigated the experiences and needs of parents with an infant with (interim) diagnosis unilateral spastic cerebral palsy with regard to pediatric physical and occupational therapy. Parents described that receiving the diagnosis of “high risk of cerebral palsy” or definite diagnosis of cerebral palsy as a devastating and overwhelming experience.^13^ Parents of infants with an (interim) diagnosis unilateral spastic cerebral palsy are motivated to engage in early intervention programs that enable parents to support neuromotor development.^13-15^ Experiences and therapy needs of parents of young children with cerebral palsy aged 2 till 4 years do not only vary between parents, but over time as well.^16^

Little is known about the experiences and therapy needs of parents of infants diagnosed with unilateral perinatal brain injury and at high risk for development of unilateral spastic cerebral palsy during the first year of life. The initial diagnostic period of unilateral perinatal brain injury and accompanying prognosis is associated with higher rates of depression, increased stress, and poor family functioning.^17,18^ On discharge from the neonatal intensive care unit, parents must wait to see if, and to what extent, their infant will develop the predicted motor deficits, which typically become apparent after 3 months. Given the sudden, unexpected, and stressful onset of unilateral perinatal brain injury, infants being at high risk for unilateral spastic cerebral palsy at our Children's Hospital are referred to a home-based community pediatric physical therapist immediately after discharge, prior to clinical signs of unilateral spastic cerebral palsy and diagnosis of unilateral spastic cerebral palsy. Additionally, as recommended in international guidelines, infants’ neuromotor development is screened on a regular basis in a hospital-based follow-up program by a multidisciplinary team, including a pediatric physical therapist and an occupational therapist. Given the uncertainty about formal diagnosis of unilateral spastic cerebral palsy, we are interested in the experiences and needs of the parents as they may be different compared with those of parents of children who have already been diagnosed with unilateral spastic cerebral palsy. The ultimate goal is to optimize pediatric and occupational therapy in guiding parents with an infant with unilateral perinatal brain injury and at high risk of unilateral spastic cerebral palsy.

Therefore, the objective of this study was to identify the experiences and needs of parents of infants with unilateral perinatal brain injury and at risk for unilateral spastic cerebral palsy based on MRI findings, with a focus on pediatric physical and occupational therapy during the first year of life. Aspects of pediatric physical and occupational therapy include neuromotor assessment and communication about the outcomes of the assessments during a hospital-based outpatient follow-up program. Home-based pediatric physical therapy included monitoring development and treatment. In infants with asymmetry in hand function, a hospital-based occupational therapist provided treatment face to face or via video call.

## Methods

### Research Design

An inductive qualitative approach was conducted to retrieve detailed experiences and needs of parents with an infant with unilateral perinatal brain injury using semistructured interviews via video calls. The Consolidated Criteria for Reporting Qualitative Research (COREQ) guideline has been used to report this study.^19^ The study was conducted in a tertiary university hospital in the Netherlands.

### Participants

Parents were eligible to participate when (1) they had an infant at risk for developing unilateral spastic cerebral palsy based on brain MRI findings, (2) they visited the outpatient follow-up clinic with their infant and received home-based pediatric physiotherapy or occupational therapy during the first year of life, (3) they spoke Dutch, and (4) their child was 2-5 years of age. Most children received a definite diagnosis of cerebral palsy by the age of 2 years. In this study, parents were asked to reflect on their experiences and needs during the first year of life of their child. Parents were included after written informed consent was obtained. This study was approved by the Medical Ethical Committee Utrecht and parents signed an informed consent for the use of data obtained from the interviews (Van Brussel/21/597-C).

### Context of Study

All included infants received standard care in the outpatient clinic of our neonatology department and received home-based physiotherapy. The outpatient clinic contains a follow-up program that aims to detect early clinical signs of unilateral spastic cerebral palsy with neuromotor assessment. In this follow-up program, a neonatologist or pediatric neurologist was involved together with a pediatric physical and occupational therapist who performed neuromotor assessments at the age of 3.5 and 9 months as recommended.^3^ At the age of 3.5 months, the General Movement Assessment ,^20^ Hammersmith Infant Neurological Examination ,^21^ and Hand Assessment for Infants ^22^ were performed. At the age of 9 months, the Hammersmith Infant Neurological Examination was conducted and in case of asymmetry on the Hammersmith Infant Neurological Examination, the Hand Assessment for Infants was performed as well. After test administration, the results were communicated with parents. In case of clinical signs of unilateral spastic cerebral palsy pediatric physical and occupational therapy was intensified with, for example, constraint-induced movement therapy.^23^

### Data Collection

A pediatric occupational therapist (CHV), who previously was involved in the care of the children, briefed parents on the study's purpose and recruited them by phone using purposive sampling (CHV and ME). Parents, both fathers and mothers, from children with and without unilateral spastic cerebral palsy and with varying severities and from diverse educational backgrounds were selected.

When parents indicated to be interested in participating in the study, written information was sent. Parents were given a 2-week period for consideration. After parents signed the informed consent, an independent social worker (MKl) who worked for 25 years in the neonatology department, conducted semistructured interviews between March 2022 and December 2022. She was trained in interview techniques, and 2 pilot interviews prior to the start of this study were performed and feedback (AvdH, CHV, and ME) was used to improve the interview.^24^ The interviewer (MKl) was not involved in treatment or neuromotor assessment of the infants. The interview guide was developed based on relevant literature and input of the full research team consisted of physicians, researchers, therapists. The interview guide comprised questions concerning experiences and needs regarding pediatric physical and occupational therapy throughout their infants’ first year of life. After 4 interviews, 2 questions were adjusted, and the interview guide was finalized. The interviews were held via video call enabling parents to stay at home and choose a convenient time and place. The interviews were recorded via MSteams and transcribed ad verbatim.

Demographic characteristics of the study participants such as age, level of parental education and gestational age of their child, and whether the infant had a definite diagnosis unilateral spastic cerebral palsy or not, were collected during the interview (Table 1). Data were collected until data saturation was reached.^25,26^ Saturation was the point when no new relevant codes were found in the data of the last 2 interviews and additional data collection would unlikely contribute substantially to a deeper understanding.

**Table 1.** Parent Participants and Their Child Demographics.

Parent				Child				
Participant number	Education^a^	Age (y)	Parent participant	Sex	Age (y;mo)	GA	Birth order	CP
P1	Medium	33	M	Male	2;8	40 ^3/7^	1	No
P2	High	42	F	Female	2;9	40	2	No
P3	High	37	M					
P4	High	38	M	Male	4;7	37 ^0/7^	2	Yes
P5	High	43	F					
P6	High	35	M	Male	3;4	37 ^3/7^	3	Yes
P7	Medium	33	M	Male	2;3	41 ^1/7^	1	No
P8	High	33	M	Male	3;11	32 ^5/7^	2	Yes
P9	Medium	34	M	Male	2;6	41 ^4/7^	3	Yes
P10	High	34	F	Female	2;0	39 ^6/7^	1	Yes
P11	High	35	M					
P12	High	35	M	Female	3;0	41 ^1/7^	2	Yes
P13	High	38	F					
P14	Medium	45	M	Female	2;1	40 ^2/7^	1	Yes
P15	Medium	43	F					
P16	High	40	F	Female	2;6	34 ^4/7^	3	Yes

Abbreviations: F, father; GA, gestational age; M, mother.

^a^ Level of education conforms to UNESCO International Standard Classification of Education (2011): Medium = ISCED 3-4; High = ISCED 5-8.

### Data Analysis

An inductive qualitative approach was used because little is known about this topic of parental experiences and needs of infants with unilateral perinatal brain injury and at risk for developing unilateral spastic cerebral palsy. Thematic analyses were iteratively applied.^27^ To familiarize with the data, the interviews were transcribed by 1 researcher (CHV) and were read several times by 2 researchers (ME and CHV). In step 2, both researchers (ME and CHV) independently generated initial codes from the first 4 interviews. They compared their codes to ensure trustworthiness before continuing to code the rest of the interviews independently. The researchers continuously reflected on and refined the coding scheme. In the next phase of searching for themes, relevant codes were grouped in themes and subthemes. In case of uncertainty in this process, the linked narratives belonging to the codes were reviewed to better understand the underlying meaning of the code. To formulate themes and subthemes, in 2 different meetings 4 researchers (MvB, AvdH, ME, and CHV) discussed, rephrased, and reordered until consensus was reached. In a third meeting (with additional researchers MKe, LdV, and JWG), themes were refined and finalized. The research team included a pediatric physical therapist (ME), a pediatric occupational therapist (CHV), 3 senior researchers among whom one is an experienced neonatal intensive care unit nurse (AvH, MKe, MvB), a pediatric rehabilitation physician (JWG), and a neonatologist (LdV). The software program NVivo (version 12.7.0) was used for the coding process. Additionally, member checking was performed by sending the participants a summary of the interview, asking if they agreed with the findings and if there were missing topics.

## Results

### Participants

Eighteen parents were contacted. One parent showed no interest, and one parent did not want to participate because of lack of time. Sixteen interviews with 10 mothers and 6 fathers from 11 infants were completed. The interviews lasted between 29 and 49 minutes. The median age of the parents was 36 years (range 33-45) and the median age of children at the time of interview was 32 months (range 24-55 months). Table 1 represents the 16 participating parents and characteristics of their infants.

All children had predominantly unilateral perinatal brain injury with involvement of corticospinal tracts confirmed on neonatal MRI and were referred to the outpatient follow-up program for being at high risk of unilateral spastic cerebral palsy. The 2 most common unilateral perinatal brain injury were perinatal arterial ischemic stroke (n = 7) and periventricular hemorrhagic infarction (n = 2); other types of injuries were antenatal periventricular hemorrhagic infarction (n = 1) and unilateral focal white matter lesions (n = 1). Two of 11 infants were born prematurely, and all children had been admitted to a neonatal intensive care unit.

All but 1 infant received community-based pediatric physiotherapy at home immediately after neonatal intensive care unit discharge, prior to clinical signs of unilateral spastic cerebral palsy. In 1 case, pediatric physiotherapy at home was started after the first visit at the outpatient clinic at 3 months of age. At the 24-month follow-up, 8 of 11 children had been diagnosed with unilateral spastic cerebral palsy. Five infants with clinical signs of unilateral spastic cerebral palsy received 1 or more periods of 6 weeks of baby-Constraint Induced Movement Therapy (baby-CIMT) by a hospital-based occupational therapist providing treatment face-to-face or via video call or a combination of both. The remaining 3 infants with clinical signs of unilateral spastic cerebral palsy received occupational therapy with a focus on bimanual training from a hospital-based occupational therapist.

### Themes

**Figure 1.**
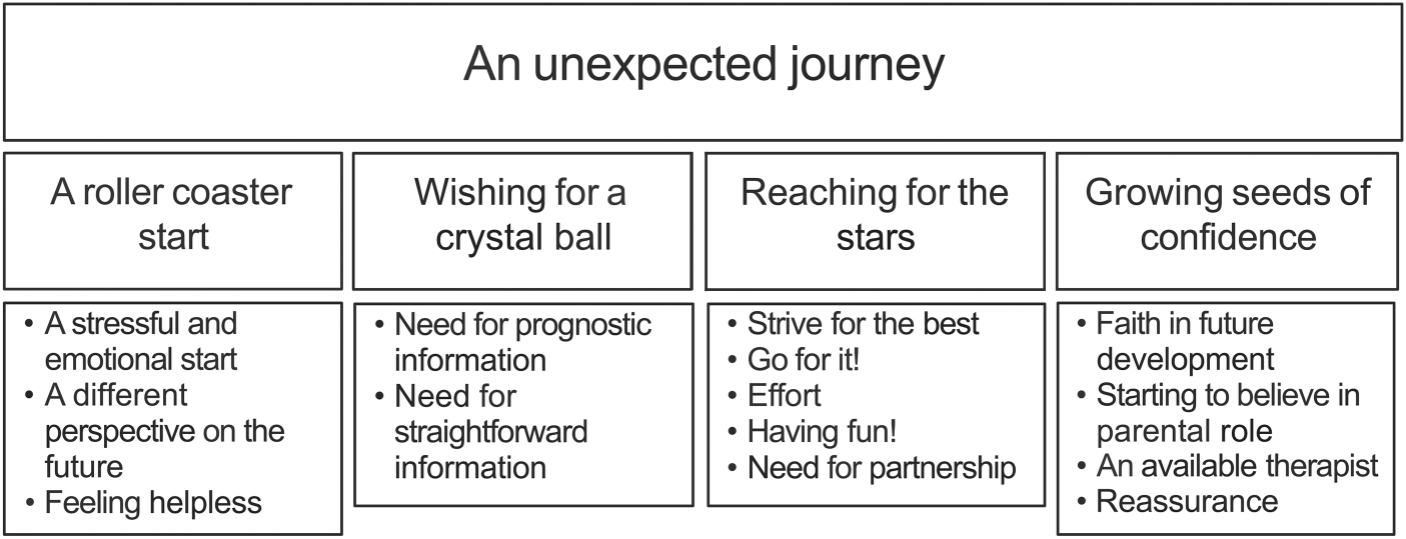
Overview of themes and subthemes.

All parents reflected on the sudden and unexpected turn in their lives with an infant with unilateral perinatal brain injury and at high risk of unilateral spastic cerebral palsy, very likely leading to a different future than anticipated. One overarching theme, “an unexpected journey,” with 4 subthemes was identified: (1) “A roller-coaster start,” (2) “Wishing for a crystal ball,” (3) “Reaching for the stars,” and (4) “Growing seeds of confidence” (Figure 1).

The first subtheme of this study, “A roller-coaster start,” comprises a theme beyond the research question as it mainly describes experiences of parents of having an infant diagnosed with unilateral perinatal brain injury and admitted to a neonatal intensive care unit. These experiences do not involve pediatric physical or occupational therapy but were a substantial part of the shared experiences with the interviewees. We decided to retain this theme for this study as it cannot be seen independently from the other themes as it serves as an introduction and provides context for the other subthemes. Quotes per subtheme can be found in Table 2.

**Table 2.** Quotes per Subtheme.

An unexpected journey		
A roller coaster start	“Yes, from the beginning, we knew it was a very severe stroke. And then maybe [name child] wouldn't be able to walk or wouldn't be able to walk for long distances, or maybe never be able to run, climb stairs, or who knows.” (P3) “You are already helpless. You can't really do anything. You can only be parents and let the specialist do the work.” (P11)	
Wishing for a crystal ball	“I needed that. Yes, that they could just look into a crystal ball and say, ‘Oh, everything will be fine,’ but yeah, it doesn't work that way. But yeah, everything they could say, what they saw, we really absorbed, so to speak.” (P4)	
Reaching for the stars	“What are the things we can do? Just tell us everything we can do to genuinely support her development as much as possible.” (P16) “The biggest need we still have, and had, uhm, is to stimulate her in that way and it doesn’t matter how much time it takes or, uhm, whether we have to go somewhere for it. But being able to do those exercises with her and help her progress. Yes, it gives the feeling that I can do something too.” (P11) “I think I can say with 100% certainty that if we hadn’t done all of that, her hand would have developed less well.” (p16) “Uhhm that there is clarity about what we're doing, why we're doing it, and which direction it's heading.” (P4)	
Growing seeds of confidence	“It’s so uncertain which direction your little one will develop, and of course, there are positive signals. But you only really know how he's doing when he shows it.” (P5) “If I couldn’t figure it out on my own, so I had no control over it, I still had control because I could still involve someone else.” (P7) “It was always somewhat limited as well by saying, yes, but do keep in mind… there are still…. ” (P14)	

#### A Roller-Coaster Start

All parents reflected on their *stressful and emotional start* with their baby. Parents described the admission as a “roller coaster” and as being overwhelmed with the diagnosis of unilateral perinatal brain injury and their infant being at high risk for development of unilateral spastic cerebral palsy. Some mothers indicated that their experiences during their stay at the neonatal intensive care unit were traumatic. Furthermore, feelings of guilt were also experienced. For instance, that seizures of the infant were not recognized in the first days of life. A parent described that he walked away during the conversation about the MRI results because it was too difficult to cope with.

All parents described that the diagnosis of unilateral perinatal brain injury and being at risk for unilateral spastic cerebral palsy confronted them with *a different perspective on the future*, which elicited feelings of anxiety and sorrow. Furthermore, parents described *feeling helpless* during the neonatal intensive care unit admission and when confronted with the diagnosis of unilateral perinatal brain injury.

#### Wishing for a Crystal Ball

Many parents wished for a “perspective” or some kind of “prediction” of future development of their infant, and for some parents this included the reassurance “that everything would be fine.” Some parents described that they were “wishing for a crystal ball.” When using the crystal ball as a metaphor, parents expressed their *need for prognostic information* about the development of their infant with unilateral perinatal brain injury. However, parents reflected that this was unrealistic because of prognostic uncertainties. Uncertainties about the severity of (future) neuromotor problems and “what it would look like” was frustrating and stressful. Parents described being dissatisfied when questions remained unanswered. Prognostic uncertainties could lead to feelings of anxiety, not knowing what to expect and what to prepare for.

Parents *needed straightforward information* that was described as “honest,” “clear,” “simple” answers to their questions about future development for their infant and in conversations about neuromotor test results. Additionally, a graphical representation of how brain damage affects motor skills was suggested. When asking for developmental outcome, parents were often confronted with possible negative scenarios. One parent was told that her child might need special education, as some children with unilateral spastic cerebral palsy do. Looking backward, information about special education was given too early and elicited mixed feelings according to one parent. Some parents experienced frustration and disappointment when they felt that a therapist avoided the conversation about future development to prevent predictive inaccuracies or raising false hope.

#### Reaching for the Stars

Many parents reported that they wanted to *strive for the best* for their infant, including “overall happiness” and “the best possible developmental outcome.” Many parents stated that guidance of a pediatric and/or occupational therapist enabled them *to go for it* and to play an active role supporting the development of their infant during the first year of life.

Parents reflected on their “transition” or adjustment from feeling helpless after receiving the diagnosis of unilateral perinatal brain injury into being able to support the development of their child. Practicing with their infant made parents feel that “they could do something.” Parents described feelings of being “satisfied” or “fulfilled” and that it gave meaning to their parental role. The flip side of doing exercises was that it took a lot of *effort* too, especially during the first months of life, and some parents were “merely surviving.” One mother did the exercises with a sense of uncertainty or from fear of doing something wrong when not doing the exercises. When looking backward, almost all parents stated that they contributed to the progress in neuromotor development of their child as early as the first weeks after diagnosis, and they felt that it was worth all the effort they put in.

Despite that doing exercises with their infant took a lot of effort, parents appreciated the playful practices with their infant. *Having fun* for both, parent and infant, is also described by parents as an important condition to prevent being overloaded as a parent and to prevent resistance of the infant, which counteracts progress in development. Some parents said having fun contributed to bonding and positive parent-infant interactions.

Parents expressed the *need for partnership* in guidance of a therapist. Parents wanted a therapist who was “humane,” “empathic,” “patient,” “passionate,” “client centered,” “had expertise about UPBI,” “did not give up,” and “strives for progress in development.” Parents described that these characteristics of their therapist were important to gain confidence in the therapist. Some parents valued the role of a therapist as a sparring partner and felt that when parents did not choose for an intervention they felt not judged. Many parents described that they wanted to know “as clearly as possible” what they could do for developmental support and why they should do it.

Knowing the background of exercises and having goals was important for shared decision making and get motivated to practice. Some parents reported that knowing and understanding the background of an exercise enabled them to apply them and fit into their daily life.

#### Growing Seeds of Confidence

Some parents reported that in advance of their visit to the outpatient clinic, they had confidence that their infant was from a neurodevelopmental point of view “on the right track,” often endorsed by the community pediatric physiotherapist. Other parents described being nervous in advance to their visit to the outpatient clinic. Nearly all parents appreciated the confirmation of improvement of development of their infant, substantiated with or without test results. Additionally, parents valued the focus on positive results and a therapist showing enthusiasm about the improvements in development. In general, parents described feelings as happiness and being proud, when infants became older and mastered milestones. Furthermore, they felt relieved that former feelings of anxiety, elicited by prognostic uncertainties of unknown neurodevelopmental disability, were ungrounded and gained *faith in future development*. This was independent of whether their infant showed signs of unilateral spastic cerebral palsy or not.

Besides growing confidence in neuromotor development of their infant, parents reported that they *started to believe in their parental role* too. Most parents described that neurodevelopmental progress of the infant reassured parents that they “were on the right track” too and that their efforts were rewarded. Developmental progress was attributed to the infant's capacity or character as well. Many parents described their infant as being “very curious,” “motivated,” and “powerful” and that this inner drive of the infant was an important requirement for neuromotor development and beyond their control. Parents felt that they supported their infants’ neuromotor development, but their infant “had to do it.” One parent of a girl who developed cerebral palsy and had multiple seizures that were hard to control reported that even the smallest and maybe doubtful progression of development was extremely important to keep hope and to carry on.

Some parents experienced frustrations during the test administration when the infant did not demonstrate its maximum competence. They appreciated a patient therapist, who considered parents’ opinion of development of their infant in the assessment. Some parents described that looking at the neuromotor assessment gave inspiration for practicing with their infant.

Additionally, parents valued that *therapists were available* when parents were uncertain about the development or had questions about anything concerning development or simply wanted to make an appointment in the hospital for follow-up. One mother experienced that having the option to ask questions to her therapist gave her feelings of being in control, knowing that she had access to support when she needed it.

Some parents of children experienced frustration that they did not get the *reassurance* from their therapist that their infant was doing well and would do well in future and still left them with uncertainties. One parent described that confirmations of a positive development frequently were accompanied with information about increased risks in development.

## Discussion

When an infant receives the diagnosis of unilateral perinatal brain injury, life takes an unexpected turn compared to what parents had envisioned for their child and a period of uncertainty begins. The current qualitative study shows that in general parents highly valued prognostic information about motor development and were motivated to support the development of their infant. Furthermore, all parents appreciated the guidance of a therapist as a partner working with the parents, which enabled them to support their infant. During the first year of life, parents gained more confidence in the development of their child and their own role; however, some parents experienced that the desired reassurance that things would continue to go well remained absent.

In line with other studies,^28^ our study showed that the impact of having an infant admitted to a neonatal intensive care unit is stressful and can even be traumatic to parents. Furthermore, receiving a diagnosis of unilateral perinatal brain injury and accompanying prognosis is an emotional and stressful experience^29-31^ and is associated with higher rates of depression.^17^ Parents in this study were prepared that their child might develop motor deficits associated with unilateral spastic cerebral palsy. During the first three months clinical signs of unilateral spastic cerebral palsy are not noticeable yet and parents still do not know exactly what to expect and how deficits in motor development and its consequences in daily functioning would look like. Parental stressors in parents with children with neonatal brain injury include thinking about long-term effects of brain injury and concerns about child's future learning abilities and independence.^32^ Although parents in this study highly valued prognostic information about future development to alleviate as much uncertainty as possible, they acknowledged also that prognostic information is limited and that uncertainty about long-term effects remains.

In general, parents in this study were motivated to support (to the maximum extent) the development of their infant. Practicing required effort, and looking backward it was worth the effort. These results are consistent with other studies in parents of young children with (interim) diagnosis unilateral spastic cerebral palsy.^13,14,33^ One parent in the current study stated that she practiced initially out of fear of doing it wrong when not practicing. Many parents were aware that because of plasticity of the brain, it is more beneficial to practice when children are young. This aligns with the “neuroplasticity sand timer effect” described by Harniess et al^34^ that can inadvertently place undue pressure on parents to practice. Other studies described the importance of positive experiences in therapy, including having fun for being motivated.^14,33,35^ In our study, parents described that having fun during practicing was not solely motivating but also protected the infant and the parent from overload and prevents parents from feeling guilty when not practicing.

Other studies described the importance of developmental progress, which gave hope and motivation to engage in therapy.^13,14,33^ In this study, both parents of infants with clinical signs of unilateral spastic cerebral palsy and those without highly valued confirmation from a therapist that their infant showed developmental progress. After discharge from the neonatal intensive care unit, parents still need to wait and see whether and to what extent their infant develops unilateral spastic cerebral palsy, mostly manifesting from 3 months onward. Parents valued a therapist, endorsed with neuromotor assessment or not, who confirmed that their child was on the right track. Even when results of a neuromotor assessment indicated signs of unilateral spastic cerebral palsy, this often confirmed the image parents already had of their child. Guttmann et al^32^ described in their study that parents remembered prognostic discussions about their children at high risk of cerebral palsy as underestimating functional outcome. It may be that the lived experience of having a young child with (clinical signs of) unilateral spastic cerebral palsy is less challenging than what parents had imagined after the discussion about the prognosis. As a result, parents in this study, including those with children with unilateral spastic cerebral palsy, may label the outcome of the neuromotor assessment as positive and predominantly confirmed that their child was doing well.

Parents’ belief about their ability to influence their child in a health- and success-promoting manner is defined as parental self-efficacy and plays an important role in the well-being of parents and their children.^36^ Guidance from a pediatric physical or occupational therapist in a collaborative partnership can reassure and empower the parental role.^35^ In this study, parents started to believe in their own role in supporting development during the first year of life. And in some cases, just having an available and easily accessible therapist is supportive for parents and contributes to the feeling of being in control. Additionally, for parents to believe in their own role, it was important that the effort in developmental support was rewarded with progress in development.

### Limitations

This study comprises a cohort of parents with children of one tertiary hospital, that is, a referral center for neonatal neurology and with extensive knowledge of brain injury and accompanying prognosis of development of unilateral spastic cerebral palsy. Parents of infants in our study received specific information about the diagnosis of unilateral perinatal brain injury and accompanying prognosis with relatively high accuracy. It can be assumed that other clinical settings do not provide such specific information, and parents might have other experiences and needs. Moreover, parents from infants without a medical history and who only after development of clinical signs of unilateral spastic cerebral palsy receive the diagnosis of unilateral perinatal brain injury, may also have different experiences and needs.

The first 2 authors with a former treatment relationship recruited parents after purposive sampling. During this process they reflected on their potential bias and discussed this with other team members to minimize it. Moreover, this study comprises parents who are relatively highly educated.

### Conclusion

Because of major advancements in MRI technology, prediction of unilateral spastic cerebral palsy is more accurate nowadays. This enables one to start with pediatric physical and occupational therapy in case of unilateral perinatal brain injury, even before clinical signs of unilateral spastic cerebral palsy arise. After a stressful start and receiving the diagnosis of unilateral perinatal brain injury, a pediatric physical therapist or occupational therapist can play a role in providing information about future (neuro)development, monitoring motor development and guidance to support a child's development. This will contribute to the needed reassurance that both parents and their infants are on the right track.

### Clinical Implications

For parents with an infant with unilateral perinatal brain injury and at high risk for unilateral spastic cerebral palsy based on MRI findings, it is desirable to focus on enablement of parents to support development, to enhance confidence in future development of their infant, and to promote parental self-efficacy. In this study, parents expressed the need for the following services: (1) individualized (prognostic) information related to the development of their child; (2) unilateral spastic cerebral palsy–specific information (written and visual); (3) easy access to a therapist who treats children and partnership of the therapist with the parents; (4) monitoring of neuromotor development of the child during the first years of life; and (5) celebrating milestones and guiding parents in supporting the development of their child.
